# The impact of childhood emotional abuse on non-suicidal self-injury in adolescents with mood disorders: a moderated mediation model

**DOI:** 10.3389/fpsyt.2025.1553437

**Published:** 2025-06-13

**Authors:** Lianzi Wang, Guifang Zha, Fangfang Chen, Jingyan Zhang, Xiaoyue Li, Ziliang Wang, Jia Luo, Chuanfu Song

**Affiliations:** ^1^ Department of Psychiatry, Wuhu Hospital of Beijing Anding Hospital, Capital Medical University(Wuhu Fourth People’s Hospital), Wuhu, Anhui, China; ^2^ National Clinical Research Center for Mental Disorders, Beijing Key Laboratory for Diagnosis and treatment of Mental Disorders, Beijing Anding Hospital, Capital Medical University, Beijing, China

**Keywords:** non-suicidal self-injury, adolescent mood disorders, childhood emotional abuse, difficulties in emotional expression, childhood trauma experiences

## Abstract

**Objectives:**

This study aims to identify factors associated with NSSI severity in adolescents with mood disorders and examine the mediating role of emotional expression difficulties and the moderating role of bullying in the relationship between childhood emotional abuse and NSSI.

**Methods:**

Using a convenience sampling method, 242 adolescents with mood disorders were surveyed with the Adolescent Self-Harm Questionnaire, Childhood Abuse Questionnaire, Toronto Alexithymia Scale, and Beck Suicidal Ideation Scale. Stepwise linear regression was performed to examine associations between NSSI severity and key predictors, including childhood trauma subtypes, alexithymia, and bullying history. The mediation and moderation effects were tested using the Hayes Process plugin.

**Results:**

The prevalence of NSSI among adolescents with mood disorders was 81.40% (n = 197). Experiences of bullying (β=0.19, P=0.001), childhood emotional abuse (β=0.25, P<0.001), and difficulties in emotional expression (β=0.25, P<0.001) were positively correlated with the severity of NSSI, while age (β=-0.12, P=0.046) was negatively correlated. Difficulties in emotional expression partially mediated the relationship between childhood emotional abuse and NSSI, with a mediation effect size of 0.25, Bootstrap 95% CI (0.02–0.10), accounting for 21% of the total effect. This mediation effect was moderated by experiences of bullying, showing a positive association between the severity of NSSI and the interaction term of emotional expression difficulties and bullying (β=3.23, P<0.001), with a moderated mediation index of 0.48 and Bootstrap 95% CI (0.08–1.14).

**Conclusion:**

Childhood emotional abuse in adolescents with mood disorders can have both direct and indirect effects on NSSI through difficulties in emotional expression, and experiences of bullying moderate the relationship between NSSI and emotional expression difficulties.

## 1 Introduction

Adolescent non-suicidal self-injury (NSSI) is a significant global public health issue, with a global detection rate of 22.1% among adolescents (1). The incidence of NSSI in clinical mental disorders ranges from 40% to 87% (2). In China, the prevalence of NSSI among adolescents varies significantly by population, ranging from 10% to 30% in community samples (3) and reaching up to 52.47% in clinical populations (4). Although NSSI is not inherently suicidal, it is strongly associated with suicidal ideation and serves as a critical risk factor for subsequent suicidal behavior (5). According to the 2019 Global Burden of Disease study, NSSI is the third leading cause of years lived with disability among individuals aged 10 to 24 (6). The factors influencing NSSI are complex and include individual characteristics, family-school-social environments, and their interactions (7). Therefore, researching the associated factors of adolescent NSSI is clinically significant, as targeted early intervention strategies based on modifiable factors can more effectively reduce the suicide risk in NSSI populations.

Current studies on adolescent NSSI have identified adverse childhood experiences (ACEs) — including physical abuse, emotional abuse, sexual abuse, and physical and emotional neglect — as significant risk factors for NSSI (8, 9). ACEs can lead to disruptions in emotional and neurophysiological functioning, resulting in NSSI and other maladaptive behaviors such as substance abuse (8). Although research has confirmed that ACEs are crucial factors influencing NSSI, the relationship between different types of ACEs and NSSI outcomes is inconsistent. Linehan’s model suggests that invalidating environments, such as those characterized by sexual abuse, contribute to emotional dysregulation and NSSI (10). However, a meta-analysis found that the effect size of the relationship between sexual abuse and NSSI is relatively small (11), and individuals with NSSI report higher rates of childhood physical abuse (12). Lang et al. (13) conducted a meta-analysis showing that early emotional abuse is a strong predictor of NSSI, while emotional neglect is most closely related to NSSI in non-clinical female samples (14). These inconsistencies may arise from the mediating and moderating factors that could influence the relationship between ACEs and NSSI, such as social support, experiential avoidance, self-esteem, pain coping styles, emotional regulation abilities, and attachment levels (7). Some studies have found a direct association between emotional abuse and NSSI (12), while the effects of sexual and physical abuse are fully mediated by emotional expression and coping.

Theoretical frameworks posit that deficits in emotional regulation constitute a core mechanism underlying NSSI (15), making the relationship between alexithymia and NSSI a focal point of research. Alexithymia is characterized by difficulties in emotional recognition and expression due to impairments in the cognitive, processing, and regulatory aspects of emotions (16), manifesting as difficulties in emotion recognition, emotion expression, and externally oriented thinking. Psychoanalytic theory suggests that alexithymia arises from early destructive events that inhibit emotional development in childhood (17). Empirical studies have shown a positive correlation between childhood trauma and alexithymia, with individuals experiencing more severe childhood trauma exhibiting higher levels of alexithymia (18, 19). Research has also found that alexithymia may increase the risk of self-harm, with difficulties in identifying emotions being the most potent predictor of self-injury (20). Although there are strong interrelations among ACEs, alexithymia, and NSSI, the psychological mechanisms underlying their interactions remain unclear. Schema theory posits that childhood emotional abuse fosters maladaptive schemas (e.g., ‘defect/shame’), which render individuals hypersensitive to subsequent stressors. Bullying, as a potent interpersonal stressor, may activate these schemas, amplifying emotional dysregulation and reinforcing reliance on maladaptive coping mechanisms like NSSI. Specifically, bullied adolescents with preexisting schemas of inadequacy or shame may perceive peer victimization as confirmation of their negative self-views, exacerbating emotional distress and impairing adaptive emotion regulation. This interplay aligns with the ‘stress sensitization’ framework, wherein early adversity heightens vulnerability to later stressors (21). Childhood trauma may impair emotional awareness and regulation (alexithymia), leaving adolescents reliant on NSSI to alleviate distress (15, 22).

In summary, this study examined the direct and indirect effects of childhood emotional abuse on NSSI severity in adolescents with mood disorders, highlighting the mediating role of emotional expression difficulties and the moderating influence of bullying. Based on schema theory and prior evidence, we hypothesized that:

1. Childhood emotional abuse would be directly associated with NSSI severity.
2. Emotional expression difficulties would mediate this relationship.
3. Bullying experiences would moderate the indirect pathway, amplifying the mediating effect.

## 2 Materials and methods

### 2.1 Objects

A convenience sampling method was used to select 242 adolescents with mood disorders from the psychological specialty outpatient clinic at Wuhu Hospital, Capital Medical University Affiliated Beijing Anding Hospital, between September 2020 and September 2021. Inclusion criteria were as follows: (1) aged 12 to 18 years; (2) diagnosed with mood disorders according to the International Classification of Diseases, 10th edition (ICD-10), with confirmation by a deputy chief physician or above; (3) able to understand the questionnaire content and respond truthfully; (4) voluntary consent from both the participant and their guardian to fill out the survey. Exclusion criteria included: (1) inability to cooperate with the evaluator due to mental and/or physical conditions; (2) co-occurring neurodevelopmental disorders; (3) history of severe alcohol or substance abuse or addiction.

In the cross-sectional study, the sample size was estimated using the formula n=(Uασδ)2n = \left (\frac{U_{\alpha} \sigma}{\delta} \right)^2n=(δUα​σ​)2 (23). Based on Lu Yueying et al.’s study, the standard deviation (σ) for NSSI severity was 20. Setting the permissible error δ to 5 and using α = 0.05 with Uα=1.96U_{\alpha} = 1.96Uα​=1.96, the calculated sample size was n=62. To account for invalid questionnaires, the sample size was increased by 20%, resulting in a final determination of at least 74 samples. Considering that the optimal sample size for mediating effect analysis should exceed 200 cases (24), this study ultimately included 242 valid samples that met the inclusion criteria. In addition to the justification for mediation analysis, we also considered the statistical power required for moderation analysis. Simulation studies by Fritz and MacKinnon suggest that sample sizes ranging from 71 to 116 are sufficient for detecting medium-sized mediated or moderated effects, depending on the analytic method. Given that our study included 242 participants, the sample size is more than adequate to support the moderation and moderated mediation analyses conducted in this research (25).

A total of 242 adolescents (M age = 15.2 years, SD = 2.1, range = 13–17; 53 boys, 189 girls) participated in the survey. The diagnoses included 57 cases of bipolar disorder, 150 cases of depressive disorder, and 35 cases of other mood disorders. Among them, 157 were urban residents and 85 were rural residents. Family structures included 134 in nuclear families, 56 in multi-generational families, 21 in single-parent families, and 27 in blended families, with 4 in other arrangements. Additionally, 74 participants reported experiences of bullying, and 221 reported having suicidal ideation (see **Supplementary Table 1**). This study has been approved by the Ethics Committee of Wuhu Hospital, Capital Medical University Affiliated Beijing Anding Hospital (Approval No. (2020)-KY-14). Cases suggesting current risk were reported to the hospital’s ethics board and, if required, to local social protection services according to national guidelines.

### 2.2 Tools

#### 2.2.1 Collection of demographic and clinical data

A self-designed questionnaire was used to collect general demographic and clinical information, including age, gender, grade, bullying history(yes/no), whether the participant is an only child, residence (rural or urban), academic performance, parents’ education level, family type, parents’ marital status, family income, diagnosis, duration of illness, and number of hospitalizations.

#### 2.2.2 Adolescent self-harm behavior questionnaire

The Chinese version of the Adolescent Self-Harm Behavior Questionnaire (ASHQ) has been validated in Chinese adolescent populations, demonstrating strong internal consistency, discriminant validity, and convergent validity (26). This questionnaire assesses the severity of NSSI by calculating the product of the frequency of self-injury and the average degree of physical harm caused (27). NSSI frequency and average harm severity are scored on a four-point scale (0 times, 1 time, 2–4 times, 5 times or more) and a five-point scale (none, mild, moderate, severe, extremely severe), respectively. The internal consistency reliability of the questionnaire is 0.85, and it demonstrates ideal discriminant validity, criterion validity, and convergent validity. In this study, Cronbach’s α coefficient was 0.953. Diagnosis of NSSI was based on the DSM-5 criteria (intentional self-harm on ≥5 days in the past year, causing moderate injury, e.g., bleeding or bruising), operationalized through self-reported frequency and severity in the Adolescent Self-Harm Questionnaire. Participants endorsing ≥5 episodes of self-harm with physical damage (scored ≥2 on severity items) were classified as meeting NSSI criteria.

#### 2.2.3 Childhood trauma questionnaire

The Chinese adaptation of the CTQ has been widely used in clinical and community samples, showing excellent reliability and factorial validity (28, 29). Comprising 28 items rated on a five-point scale, this questionnaire includes five factors: emotional abuse, physical abuse, sexual abuse, emotional neglect, and physical neglect (30). The Chinese version of the CTQ has shown good reliability and validity. In this study, Cronbach’s α coefficient was 0.861.

#### 2.2.4 Toronto alexithymia scale

The Chinese version was translated by Zhu (31), and the TAS-20 (Chinese version) demonstrates high internal coherence, reliability test-record, and convergent validity (32). This scale consists of 20 items rated on a five-point scale and includes three factors: difficulties in emotion recognition, difficulties in emotion expression, and externally oriented thinking. Higher scores indicate a greater level of alexithymia (33). In this study, Cronbach’s α coefficient was 0.861.

#### 2.2.5 Beck scale for suicide ideation-Chinese version

The Beck Scale for Suicide Ideation-Chinese Version (BSI-CV) has been validated in Chinese adolescents, showing high internal consistency and strong criterion validity against clinician-rated suicidality (34). This scale uses a three-point scoring system (0-2). Participants first complete the first five items; if both items 4 and 5 are answered as “no,” it is considered that there are no suicidal ideations, and the questionnaire ends. If either item 4 or 5 is answered as “weak” or “moderate to strong,” it is determined that there are suicidal ideations, and the participant must continue with the remaining 14 items to assess suicidal risk (35). This study used the first five items to evaluate suicidal ideations over the past week and during the lowest point of depression; a higher total score indicates stronger suicidal ideations. In this study, Cronbach’s α coefficient was 0.898. Sample Items from Measurement Scales see **Supplementary Material**.

### 2.3 Quality control

Trained researchers explained the purpose and significance of the study to the participants. All participants volunteered and provided informed consent, signed by both the participant and their guardian. Demographic data were obtained through one-on-one interviews, while self-assessment scales were completed independently by the patients and collected on-site afterward. Questionnaires with errors or those completed too quickly were deemed invalid. A total of 250 questionnaires were distributed, with 8 invalid questionnaires removed, resulting in an effective recovery rate of 96.8%.

### 2.4 Statistical methods

Data were analyzed descriptively using SPSS 26.0 software, and common method bias was tested. A significance level of P < 0.05 was considered statistically significant. An unrotated exploratory factor analysis showed that the first factor explained 21.52% of variance, below the 40% threshold (24), indicating no significant common method bias. A stepwise linear regression model was constructed to explore the associated factors of NSSI severity. To validate the robustness of predictors identified through stepwise regression and control for potential confounders, a hierarchical linear regression was subsequently performed in **Supplementary Material**. The mediation and moderation effects of the NSSI severity regression model in adolescents with mood disorders were tested using models 4 and 14 in the process 4.1 developed by Hayes; Bootstrap tests with a 95% confidence interval that did not include 0 indicated significant results (36).

## 3 Results

### 3.1 Common method bias test

Following recommendations from related research, quality control measures were implemented in the measurement procedures, and Harman**’**s single-factor method was used to test for common method bias. The number of common factors was set to 1, with 22 factors having eigenvalues greater than 1. The variance explained by the first common factor was 21.52%, which is significantly below the critical standard of 40%. Therefore, this study does not exhibit common method bias.

### 3.2 Prevalence of NSSI in adolescents with mood disorders

According to the DSM-5 diagnostic criteria for NSSI, individuals must have engaged in intentional self-harm on five or more days in the past year, causing moderate bodily injury such as bleeding, bruising, or pain. The proportion of participants meeting these NSSI diagnostic criteria in this study was 81.40% (197 cases).

### 3.3 Stepwise linear regression analysis of factors influencing NSSI severity in adolescents with mood disorders

Using NSSI severity as the dependent variable, demographic variables such as gender, age, experiences of bullying, residence, and family background were analyzed alongside the total scores from the BSI-CV, CTQ, and its factors (emotional abuse, physical abuse, sexual abuse, emotional neglect, and physical neglect), as well as the total scores from the TAS and its factors (difficulties in emotion recognition, difficulties in emotion expression, and externally oriented thinking). The final model included four variables: “age, history of bullying, CTQ emotional abuse score, and TAS emotional expression difficulty score,” which explained 24.5% of the variance in NSSI severity (R² = 0.24, F = 19.21, P < 0.001). History of bullying, CTQ emotional abuse score, and TAS emotional expression difficulty score were significant positive predictors of NSSI severity in adolescents with mood disorders, while age was a significant negative predictor (see **Table 1**). Notably, CTQ-emotional abuse showed a strong positive correlation with NSSI (r = .382, p <.001), as did difficulties in emotion recognition (r = .317, p <.001) and expression (r = .315, p <.001). Physical neglect also correlated moderately with NSSI (r = .248, p <.001). Sexual abuse did not correlate significantly with NSSI (r = .072, p = .265), highlighting the specificity of emotional forms of trauma and alexithymia domains in relation to self-injury (See **Supplementary Table 2**). Importantly, these findings are highly consistent with the results obtained using hierarchical regression, supporting the robustness of the identified associations and the central role of emotional abuse and emotional expression difficulties in adolescent NSSI (see **Supplementary Table 3**).

**Table 1:** Results of the stepwise linear regression analysis on the severity of NSSI (n=242).

	Unstandardized Coefficients		Standardized Coefficients	t-value	P-value	Collinearity Diagnostics	
	*B*	*SE*	*β*			VIF	Tolerance
Age	-1.94	0.97	-0.12	-2.00	0.046*	1.08	0.93
Bullying History	11.28	3.40	0.19	3.32	0.001**	1.06	0.94
TAS - Difficulties in Emotion Expression	1.81	0.43	0.25	4.21	<0.001**	1.08	0.92
CTQ - Emotional Abuse Score	1.24	0.30	0.25	4.08	<0.001**	1.19	0.84
R^2^	0.24						
Adjusted R²	0.23						
*F*	*F*(4,237)=19.21,*P*<0.001						
D-W Value	2.17						

*P<0.05, **P<0.01, ***P<0.001.

### 3.4 Moderated mediation analysis of bullying history in the regression model of emotional abuse, difficulties in emotion expression, and NSSI severity

**Figure 1:**
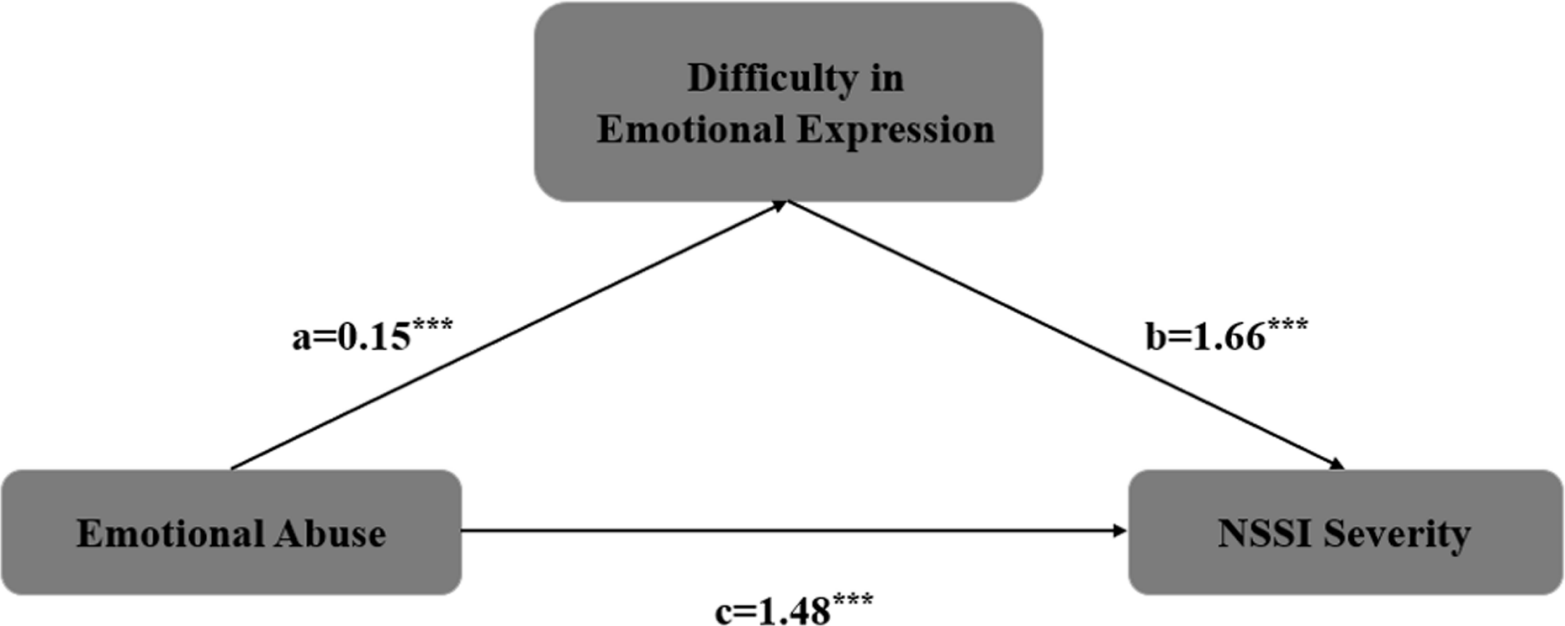
The mediating effect of emotional expression difficulties on the relationship between emotional abuse and the severity of NSSI ***P<0.001.

After controlling for age, the CTQ emotional abuse score, TAS difficulties in emotion expression score, and NSSI severity score were simultaneously entered into Model 4. The results showed a positive association between the TAS difficulties in emotion expression score and the CTQ emotional abuse score (a = 0.15, SE = 0.04, P < 0.001); a positive association was also found between the NSSI severity score and the CTQ emotional abuse score (c’ = 1.48, SE = 0.30, P < 0.001); furthermore, the NSSI severity score was positively associated with the TAS difficulties in emotion expression score (b = 1.66, SE = 0.44, P < 0.001). The Bootstrap analysis results indicated that a × b = 0.25, Boot SE = 0.02, with a 95% CI of (0.02 to 0.10) that does not include 0, suggesting that the TAS difficulties in emotion expression score plays a partial mediating role between the CTQ emotional abuse score and NSSI.The mediation effect accounted for 21% of the total effect. Detailed results are shown in **Tables 2**, **3** and **Figure 1**.

**Table 2:** Partial mediating effect of difficulty in emotional expression between emotional abuse and NSSI severity.

Independent Variable	Dependent Variable	*Ｒ^2^*	*F-*value	*β*	*SE*	t-value
CTQ-Emotional Abuse Score	TAS-Difficulty in Emotional Expression Score	0.07	8.43***	0.22	0.04	3.41
TAS-Difficulty in Emotional Expression Score	NSSI Severity			0.35	0.30	5.72
CTQ-Emotional Abuse Score	NSSI Severity	0.21	21.05***	0.30	0.30	4.92

***P<0.001

**Table 3:** Test of partial mediating effect of difficulty in emotional expression between emotional abuse and NSSI severity.

Effect	Path	Effect Value	*SE*	*Boot LLCI*	*Boot ULCI*
Direct Effect	Emotional Abuse ⇒ NSSI Severity	1.48	0.30	0.89	2.08
Indirect Effect	Emotional Abuse ⇒ Difficulty in Emotional Expression	0.15	0.04	0.06	0.24
	Difficulty in Emotional Expression ⇒ NSSI Severity	1.66	0.44	0.80	2.51
	Emotional Abuse⇒Difficulty in Emotional Expression ⇒ NSSI Severity	0.25	0.02	0.02	0.10
Total Effect	Emotional Abuse ⇒ NSSI Severity	1.731	0.30	1.14	2.33

***P<0.001.

Controlling for age, four variables—CTQ emotional abuse score, TAS difficulty in emotional expression score, NSSI severity, and bullying history—were included in Model 14, with bullying history as a moderator and difficulty in emotional expression as a mediator. The results showed that the interaction between NSSI severity, TAS difficulty in emotional expression score, and bullying history was positively associated (β = 3.23, P < 0.001), with a moderated mediation index of 0.48 and a 95% Bootstrap confidence interval (CI) of 0.08–1.14, indicating that the moderated mediation model was valid. Specifically, when bullying levels were low, the mediation effect of TAS difficulty in emotional expression score was not significant (effect size = 0.04, 95% Bootstrap CI = -0.16 to 0.23); at moderate bullying levels, the mediation effect was significant (effect size = 0.26, 95% Bootstrap CI = 0.08 to 0.52); and at high bullying levels, the mediation effect was also significant (effect size = 0.48, 95% Bootstrap CI = 0.14 to 0.98), as shown in **Tables 4**, **5**.

**Table 4:** Moderated mediation effect test of emotional abuse and NSSI severity in adolescents with mood disorders.

	NSSI Severity			Difficulty in Emotional Expression		
	β	SE	t	β	SE	t
Constant	63.45	28.25	2.25*	19.55	2.63	7.42***
CTQ Emotional Abuse Score	1.27	0.30	4.30***	0.15	0.04	3.41***
Bullying	-47.50	16.00	-2.97***			
TAS Difficulty in Emotional Expression Score	-2.48	1.22	-2.04*			
TAS Difficulty in Emotional Expression * Bullying	3.23	0.86	3.75***			
R^2^	0.29			0.07		
F-Value	19.03***			8.43***		

* *P*<0.05, ** *P*<0.01, *** *P*<0.001.

**Table 5:** Moderation effect test of bullying history in the relationship between emotional abuse and NSSI severity.

Mediator Variable	Level of Bullying	Value of Effect	SE	Boot LLCI	Boot ULCI	Index	Boot SE	Boot LLCI	Boot ULCI
TAS Difficulty in Emotional Expression Score	Low Level (-1SD)	0.04	0.10	-0.16	0.23	0.48	0.28	0.08	1.14
	Average Level	0.26	0.11	0.08	0.52				
	High Level (+1SD)	0.48	0.22	0.14	0.98				

## 4 Discussion

This study examined the factors influencing NSSI severity in adolescents with mood disorders, finding positive correlations between NSSI severity and experiences of bullying, childhood emotional abuse, and difficulties in emotional expression, while age was negatively correlated with NSSI severity. Additionally, the study explored the relationships among childhood emotional abuse, difficulties in emotional expression, and NSSI, revealing that childhood emotional abuse not only directly affects NSSI but also exerts an indirect effect via difficulties in emotional expression. Furthermore, this influence is moderated by bullying history, with bullying experiences enhancing the positive predictive effect of emotional expression difficulties on NSSI.

In a cross-sectional survey of 242 adolescent patients with mood disorders, the prevalence of NSSI was 81.40%, which is higher than the NSSI prevalence reported in previous clinical studies within China (37). However, a cross-sectional study abroad on adolescent inpatients found an even higher NSSI prevalence of 87% (38). The variation in NSSI prevalence across studies may stem from differences in sample sources, inclusion criteria, and illness severity. The observed NSSI prevalence of 81.4% in outpatient mood disorder clinics underscores the critical need for systematic NSSI screening at both initial intake and routine follow-up visits. In light of the high levels of severe psychopathology, extended illness duration, and frequent comorbidity with suicidal ideation observed in our sample, we recommend the incorporation of validated self-harm screening instruments—such as the Adolescent Self-Harm Questionnaire—into standard clinical protocols. Early identification of self-injurious behaviors allows for prompt referral to targeted interventions, including skills-based trainings in emotion regulation, distress tolerance, and safety planning. Furthermore, implementing integrated psychoeducation programs for patients and their families can enhance awareness of NSSI risk factors, reduce stigma, and foster timely help-seeking. By embedding these practices into outpatient services, clinicians can more effectively mitigate self-harm behaviors and improve overall treatment outcomes in this high-risk adolescent population.

Among the 242 patients included in this study, 57 were diagnosed with bipolar disorder, 150 with depressive disorders, and 59.31% had been ill for more than a year. Additionally, 91.30% of participants reported suicidal ideation, indicating a relatively severe sample. The high prevalence may also relate to the study design, as convenience sampling was used, which may introduce selection bias. Future research could address this limitation by conducting a probability sampling survey through a multicenter national study to investigate NSSI prevalence among adolescents with mood disorders across China.

This study found that age significantly negatively predicts the severity of NSSI in adolescents with mood disorders. Adolescence is a critical developmental period characterized by a mismatch in the development of the limbic system and the prefrontal cortex neural networks, which can lead to heightened impulsivity and emotional reactivity. The limbic system, responsible for driving emotions, undergoes rapid development during puberty, while the prefrontal cortex, which is primarily responsible for rational judgment and impulse control, develops relatively later (39). A systematic review by Plener et al. (40) of 32 longitudinal studies on self-harming behaviors indicated that NSSI shows an upward trend in early adolescence, peaks during puberty (ages 15-17), and gradually declines in late adolescence or early adulthood. In this study, no significant effect of gender on NSSI was observed, despite some research indicating that the prevalence of NSSI is higher in females. Currently, the relationship between NSSI and gender remains unclear.

While there is a close relationship between adverse childhood experiences and NSSI, this study found that only emotional abuse among these experiences significantly positively predicted NSSI, consistent with some findings both domestically and internationally (37). Childhood emotional abuse refers to long-term exposure to a highly critical, controlling, or even hostile family environment during early years, which conveys messages to children that they are “unlovable, worthless, or even in danger” (37). Meta-analyses have shown that emotional abuse is most closely related to young schema theory compared to other subtypes of childhood adversity (41). Schema theory posits that children who suffer emotional abuse have unmet core needs, making them more likely to develop maladaptive coping mechanisms such as NSSI when encountering stressors during their development (42). Research on different types of trauma experiences has shown that emotional abuse and emotional neglect are associated with an increased risk of NSSI (8, 12, 43). After adjusting for age and gender, emotional abuse has been identified as an independent risk factor for NSSI. Although physical and sexual abuse are characterized by acute trauma, emotional abuse is linked to more severe and long-term negative outcomes. It can serve as a foundation for the effects of other forms of childhood abuse and exacerbate their impact (21).

The results of this study indicate that childhood emotional trauma not only directly affects non-suicidal self-injury (NSSI) but also indirectly influences NSSI through difficulties in emotional expression associated with alexithymia. Childhood emotional abuse can impact children’s understanding of emotions, leading to a decreased ability to identify and differentiate emotions, which in turn triggers alexithymia (22). Previous research has confirmed that alexithymia, particularly difficulties in emotional recognition and expression, is closely related to NSSI (20). In this study, difficulties in emotional recognition and externally oriented thinking within alexithymia did not show significant predictive power for NSSI; however, difficulties in emotional expression were found to significantly positively predict NSSI. International studies have identified emotional recognition difficulties as the strongest predictor of NSSI (20). The discrepancy in results may be related to differences in sample selection; the previous studies were based on student populations, while this study focused on patients with mood disorders, suggesting that the associated factors for NSSI may vary across different groups.

While this study focused on childhood emotional abuse as a key predictor of NSSI, we acknowledge that adolescents often experience multiple forms of maltreatment (e.g., physical abuse, neglect), and cumulative trauma may exert a dose-response effect on NSSI risk. Prior research highlights that the accumulation of adverse childhood experiences (ACEs) significantly amplifies the likelihood of maladaptive outcomes, including emotion dysregulation and self-injury (43). For instance, a meta-analysis by Liu et al. (2018) demonstrated that each additional ACE increases the odds of NSSI by 25% (9). In our sample, preliminary correlations (**Supplementary Table 2**) revealed moderate associations between other trauma subtypes (e.g., physical abuse, neglect) and NSSI severity, though emotional abuse emerged as the strongest individual predictor. This aligns with schema theory, which posits that emotional abuse uniquely undermines core emotional needs (e.g., safety, self-worth), creating a foundation for maladaptive coping (21).

This study also found that the impact of difficulties in emotional expression on NSSI is moderated by experiences of bullying, where bullying experiences enhance the positive predictive effect of emotional expression difficulties on NSSI. Previous research has established a link between bullying behaviors and the risk of NSSI in adolescents (22, 44); bullied adolescents are 2.41 times more likely to engage in NSSI compared to their non-bullied peers. It is suggested that adolescents may resort to NSSI as a way to alleviate the negative emotions caused by bullying and to gain social support and a sense of self-control (45). According to schema theory, emotional abuse can create maladaptive schemas of “defect/shame,” which can be activated when individuals encounter stressors such as bullying, leading to a series of negative emotions (21). When individuals find it difficult to describe and express their negative emotions, they may resort to coping strategies such as NSSI.The moderating role of bullying aligns with schema theory’s emphasis on stressor activation. Adolescents with histories of emotional abuse may internalize maladaptive schemas that distort their interpretation of bullying experiences (e.g., interpreting teasing as evidence of inherent unworthiness). This cognitive-emotional cascade may intensify reliance on NSSI as a means of externalizing unarticulated distress or regaining control. Importantly, bullying may also reduce opportunities for social support—a protective factor against NSSI—thereby compounding the indirect pathway through emotional expression difficulties.

However, this study also has some limitations. For instance, it is a cross-sectional retrospective study, which may introduce certain recall and emotional biases, especially regarding childhood trauma experiences. The cross-sectional design precludes conclusions about the temporal sequence of emotional abuse and bullying. Future longitudinal studies are needed to validate these pathways. Our mediation model did not test cumulative trauma due to methodological constraints. Stepwise regression prioritized emotional abuse for its statistical significance, but this approach may overlook synergistic effects of co-occurring adversities. For example, emotional abuse coupled with bullying—a common stressor in adolescence—may exacerbate alexithymia and NSSI risk beyond the impact of single traumas. Future studies should adopt cumulative risk models to quantify the additive or interactive effects of multiple traumas. This would align with the “dose-response” framework and inform more holistic interventions targeting poly victimized youth. The measurement of bullying relied on a single yes/no item, limiting our ability to capture its frequency, severity, or forms (e.g., physical, verbal, cyber). This binary operationalization may underestimate the nuanced impact of bullying and reduce statistical power. Future studies should employ validated multidimensional scales (e.g., the Olweus Bullying Questionnaire) to assess bullying more comprehensively. Additionally, while our findings highlight emotional abuse as a critical predictor, the cross-sectional design and focus on single trauma types limit insights into cumulative trauma effects. Adolescents often endure multiple adversities, and future longitudinal studies should test whether cumulative trauma mediates or moderates NSSI pathways more robustly than individual subtypes. Future research could utilize longitudinal data to deepen verification. Additionally, as the sample was drawn from a single hospital, prospective studies with a larger sample size and broader scope could further explore the mechanisms and risk factors associated with NSSI, leading to the development of more effective prevention and intervention measures.

Non-suicidal self-injury (NSSI) is a typical and serious maladaptive behavior, and finding thorough and effective interventions poses a challenge and a key focus in clinical work. The results of this study provide clear insights for the prevention and psychological intervention of NSSI in adolescents with mood disorders. Childhood emotional abuse and bullying, as risk factors that can be identified and modified early, hold significant value for the assessment and intervention of NSSI. In the future, routine clinical screening for adverse childhood experiences and bullying among adolescents with mood disorders can be implemented. Based on the assessment results, appropriate training in emotional recognition, expression, and regulation skills can be provided, thereby effectively preventing and treating NSSI in this population.

## Data Availability

The original contributions presented in the study are included in the article/**Supplementary Material**. Further inquiries can be directed to the corresponding authors.
