# Supplementary material for: The impact of childhood emotional abuse on non-suicidal self-injury in adolescents with mood disorders: a moderated mediation model
Source: Front Psychiatry. 2025 Jun 13;16:1553437. doi: 10.3389/fpsyt.2025.1553437 (PMC12202515; doi:10.3389/fpsyt.2025.1553437)
Supplement: Supplementary file 1 [file DataSheet1.pdf]

Supplement Table 1. Participant Characteristics (N = 242)

**Variable**	**Category**	**n (%)**
Gender	Male	53 (21.9%)
	Female	189 (78.1%)
Residence	Urban	68 (28.1%)
	County Town	89 (36.8%)
	Rural	85 (35.1%)
Grade	Primary School	6 (2.5%)
	Middle School	111 (45.9%)
	High School	115 (47.5%)
	University	10 (4.1%)
Bullying History	Yes	74 (30.6%)
	No	168 (69.4%)
Family Type	Nuclear	134 (55.4%)
	Multigenerational	52 (21.5%)
	Skipped-generation	4 (1.7%)
	Single-parent	21 (8.7%)
	Blended	27 (11.2%)
	Other	4 (1.7%)
Diagnosis	Depressive Disorder	189 (78.1%)
	Bipolar Disorder	14 (5.8%)
	Other Mood Disorders	39 (16.1%)

Supplement Table2 Correlation Matrix of Key Study Variables

Variable	the severity of NSSI	CTQ-emotional abuse	CTQ-physical abuse	CTQ-sexual abuse	CTQ-emotional neglect	CTQ-physical neglect	TAS-difficulties in emotion recognition	TAS-difficulties in emotion expression	TAS-externally oriented thinking
1. the severity of NSSI	—								
	—								
2. CTQ-emotional abuse	0.382	—							
	< .001	—							
3. CTQ-physical abuse	0.188	0.624	—						
	0.003	< .001	—						
4. CTQ-sexual abuse	0.072	0.187	0.292	—					
	0.265	0.003	< .001	—					
5. CTQ-emotional neglect	0.237	0.42	0.301	-0.001	—				
	< .001	< .001	< .001	0.986	—				
6. CTQ-physical neglect	0.248	0.47	0.421	0.299	0.605	—			
	< .001	< .001	< .001	< .001	< .001	—			
7. TAS-difficulties in emotion recognition	0.317	0.313	0.076	-0.041	0.095	0.096	—		
	< .001	< .001	0.236	0.521	0.142	0.136	—		
8. TAS-difficulties in emotion expression	0.315	0.242	0.077	-0.094	0.137	0.102	0.763	—	
	< .001	< .001	0.235	0.146	0.034	0.113	< .001	—	
9. TAS-externally oriented thinking	0.097	0.096	0.013	0.073	0.146	0.064	0.186	0.192	—
	0.131	0.135	0.838	0.257	0.024	0.323	0.004	0.003	—

CTQ = Childhood Trauma Questionnaire; TAS = Toronto Alexithymia Scale; NSSI = Non-Suicidal Self-Injury

The matrix presents Pearson's r coefficients and p-values for associations among CTQ subscales (emotional abuse, physical abuse, sexual abuse, emotional neglect, physical neglect), TAS factors (difficulties in emotion recognition, difficulties in emotion expression, externally oriented thinking), and NSSI severity. Notably, CTQ-emotional abuse showed a strong positive correlation with NSSI ($r = .382$, $p < .001$), as did difficulties in emotion recognition ($r = .317$, $p < .001$) and expression ($r = .315$, $p < .001$). Physical neglect also correlated moderately with NSSI ($r = .248$, $p < .001$). Sexual abuse did not correlate significantly with NSSI ($r = .072$, $p = .265$), highlighting the specificity of emotional forms of trauma and alexithymia domains in relation to self-injury.

Supplement Table 3 Results of the Hierarchical Regression Analysis on the Severity of NSSI(n=242).

	**Unstandardized Coefficients**		**Standardized Coefficients**	**t-value**	**P-value**	**Collinearity Diagnostics**	
	B	***SE***	***β***			**VIF**	**Tolerance**
**Bullying History**	12.27	3.47	0.21	3.54	<0.001***	1.09	0.91
**TAS - Difficulties in Emotion Expression**	1.40	0.66	0.19	2.14	0.033*	1.08	0.92
**CTQ - Emotional Abuse Score**	0.96	0.43	0.19	2.21	0.028*	2.38	0.42
**R^2**	0.27						
**Adjusted R^2**	0.23						
F	$F(12,229)=7.05, P<0.001$						
**D-W Value**	1.943						

*P<0.05, ***P<0.001

To explore factors associated with NSSI severity, a hierarchical linear regression model was conducted. NSSI severity was used as the dependent variable. In Block 1, control variables—gender, age, experiences of bullying, residence, and family background—were entered. In Block 2, the total scores from the BSI-CV, CTQ, and its factors (emotional abuse, physical abuse, sexual abuse, emotional neglect, and physical neglect), as well as the total scores from the TAS and its factors (difficulties in emotion recognition, difficulties in emotion expression, and externally oriented thinking) were entered simultaneously to examine their unique contributions.

The final model explained 27% of the variance in NSSI severity ($R^2 = 0.27$, Adjusted $R^2 = 0.23$, $F(12,229) = 7.05$, $P < 0.001$). Bullying history ($\beta = 0.21$, $P < 0.001$), CTQ emotional abuse ($\beta = 0.19$, $P = 0.028$), and TAS difficulties in emotion

expression ($\beta = 0.19$, $P = 0.033$) emerged as significant positive predictors. Other trauma and alexithymia dimensions did not reach significance. Notably, age—previously significant in the stepwise model—was no longer statistically significant in the hierarchical regression after accounting for emotional and emotional-processing variables.

Sample Items from Measurement Scales

1. Childhood Trauma Questionnaire (CTQ)

Sample Items (5-point Likert scale: 1 = "Never True" to 5 = "Very Often True")

Emotional Abuse:

"I felt that my family hated me."

"People in my family called me things like 'stupid' or 'lazy'."

Physical Abuse:

"I was punished with a belt, a board, or some other hard object."

Sexual Abuse:

"Someone tried to touch me in a sexual way or make me touch them."

Emotional Neglect:

"I felt loved in my family." (Reverse-scored)

Physical Neglect:

"I didn't have enough to eat."

2. Toronto Alexithymia Scale (TAS-20)

Sample Items (5-point Likert scale: 1 = "Strongly Disagree" to 5 = "Strongly Agree")

Difficulty Identifying Feelings (DIF):

"I am often confused about what emotion I am feeling."

Difficulty Describing Feelings (DDF):

"It is difficult for me to find the right words for my feelings."

Externally Oriented Thinking (EOT):

"I prefer talking to people about their daily activities rather than their feelings."

3. Adolescent Self-Harm Questionnaire (NSSI Assessment)

Sample Items

Frequency (4-point scale: 0 = "Never" to 3 = "5+ times")

"In the past year, how often have you deliberately hurt yourself (e.g., cutting, burning) without suicidal intent?"

Severity (5-point scale: 0 = "No injury" to 4 = "Severe injury")

"On average, how physically damaging were these acts?" (Examples: minor scratches, bleeding, scarring)

4. Beck Scale for Suicide Ideation (BSI-CV)

Sample Items (3-point scale: 0 = "No" to 2 = "Strong")

"I have thoughts of killing myself but would not carry them out."

"I have a specific plan for suicide."
